# Harnessing the Therapeutic Potential of Exosomes: A Novel Strategy for Anticancer and Antiviral Therapy

**DOI:** 10.1155/2022/3356467

**Published:** 2022-09-12

**Authors:** Njinju Asaba Clinton, Nkembi-Leke Joshua Ageboh, Baie Decler Nkache, Ebamu Sylvia Mencha, Asonganyi Aminkeng, Ewalu Justa Ndobegang, Esembieng Mencha Ivo, Simaa Rene Vigha, Cyril Jabea Ekabe

**Affiliations:** ^1^Grace Community Health and Development Association, P. O. Box, 15 Kumba, Southwest Region, Cameroon; ^2^Health and Empowerment Foundation, Cameroon; ^3^Faculty of Health Sciences, University of Buea, Cameroon; ^4^Mbonge District Hospital, South West Region, Cameroon

## Abstract

Exosomes are extracellular membrane bound vesicles released from almost all cell types and can be retrieved from all body fluids. The molecular constituents of these extracellular bodies vary depending on their cell of origin, from which they can transport molecules such as DNA, RNA, proteins lipids, and several metabolites. They have been shown to execute several functions such as in cell growth, migration, differentiation, neuronal signaling, immune cell modulation, and some diseases such as cancer through intercellular communication and signaling. They are also described to act as key players in viral persistence and dissemination. Due to their ability to elicit potent cellular responses, high level of tolerance in host cells, and high efficiency in penetrating other cells, they are proposed to be potential therapeutics as well as vehicles for drug delivery. In recent years, several studies have been conducted in quest for the development of an effective anticancer therapy or antiviral therapy against highly persistent viruses. However, most of these studies become halted due to failure to achieve desired therapeutic outcomes. Nevertheless, the *in vitro/in vivo* application of exosomes in tumor and infectious disease diagnosis and therapy is prospective. This review discusses the role of exosomes as predictive markers for immune activation and potential targets for anticancer/antiviral therapies.

## 1. Introduction

Exosomes are extracellular membrane bound vesicles of approximately 40-120 nm released from almost all cell types and can be retrieved from all body fluids [1, 2]. They were described as early as in the 1980s [3] from maturing mammalian reticulocytes and are formed from multivesicular bodies (MVBs), generated by the inward budding of endosomal membrane which fuse with the plasma membrane to ensure their deposition into the extracellular space [4]. The molecular constituents of these extracellular bodies vary depending on their cell of origin, from which they can transport molecules such as DNA, RNA, proteins lipids, and several metabolites [5]. Perhaps, they represent the complexity of their precursor cells and display intrinsic abilities to control complex biological function in both physiological and pathological conditions. They have been shown to execute several functions such as in cell growth, migration, differentiation, neuronal signaling, immune cell modulation, and some diseases such as cancer through intercellular communication and signaling [1]. They are described to be key players in triggering viral persistence and dissemination by harboring viral nucleic acids or transfer of proteins/virus specific receptors that make cells more susceptible to viral infection [6]. Studies have shown that these nanovesicular bodies can express a variety of surface molecules including the MHC molecules, thus enabling them to execute some immune-related functions. Moreover, it has been shown that MHC II expressing exosomes attached on the surface of follicular dendritic cells (FDCs) provided a source of continuous activation of specific B lymphocytes through the presentation of processed antigens in the form of peptide-loaded MHC class II [7]. In addition, reports have shown that exosomes isolated from activated human monocyte derived DCs could stimulate peripheral CD8 T cells in vitro, independently of target DCs, demonstrating an intrinsic stimulatory capacity of exosomes [4, 8, 9]. Despite the ability of these nanovesicles in inducing protective immune responses, they can also favor pathogen immune evasion and thus prevent disease clearance [10]. Emerging evidence highlights their pathological role in HIV infection through the transfer of contents of the HIV Nef protein or the C-C chemokine receptors (CCR5) which could trigger the reactivation of viral replication in latent cells or render naive cells susceptible [11, 12]. The unique and complex composition of exosomes provides a multicomponent diagnostic window for early disease detection and monitoring. Due to their presence in all body fluids, this provides sample diversity as well as minimally invasive sample collection procedures for a continuous and long-term monitoring of patient response to a therapy over the course of treatment [13]. Due to the ability of exosomes to elicit potent cellular responses, high level of tolerance in host cells, and high efficiency in penetrating other cells, they are proposed to be potential therapeutics as well as vehicles for drug delivery. Some exosome-based vaccines such as dendritic cell-derived, tumor cell-derived, and ascitic cell-derived exosome-based vaccines are currently under development.

In recent years, several studies have been conducted in quest for the development of an effective anticancer therapy or antiviral therapy against highly persistent viruses. However, the majority of these studies have been halted due to failure to achieve desired therapeutic outcomes. Nevertheless, the *in vitro/in vivo* application of exosomes in tumor and infectious disease diagnosis and therapy is prospected. This review discusses the role of exosomes as predictive markers for immune activation and potential targets for anticancer/antiviral therapies.

## 2. Molecular Mechanisms of Exosomes Biogenesis and Composition

**Figure 1:**
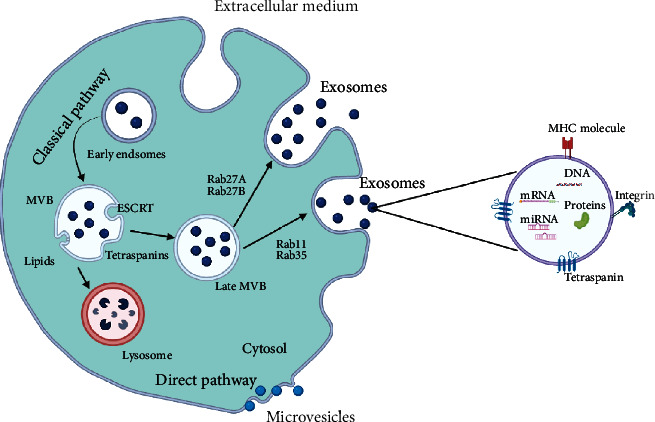
Schematic illustration of the various pathways involved in exosome formation and release, including some essential molecules which form the composition of exosomes. The classical pathway of exosome biosynthesis comprises the endocytosis pathway, while in the direct pathway, exosomes are synthesized directly from the plasma membrane.

Several mechanisms have been recognized for the biogenesis of exosomes, but much remains to be understood with regards to some of these mechanisms [14]. There are two pathways through which exosomes are synthesized: either the classic or the direct pathways [15]. Both pathways for exosome synthesis involve the processes of formation, cargo sorting, secretion, and uptake [16]. The classical pathway of exosome biosynthesis comprises the endocytosis pathway, while in the direct pathway, exosomes are synthesized directly from the plasma membrane and this often happens in T cells (Figure 1) [17].

The first step of exosome biogenesis involves the inward budding of the lipid raft domains of the plasma membrane to form endocytic vesicles, which lead to the intracellular formation of early endosomes [14, 18]. The early endosomes develop into late endosomes with the help of the Golgi complex [19]. During this process, intraluminal vesicles (ILVs) accumulate in their lumen via the process of the inward invagination of the early endosomal membrane and cytosol sequestration. Furthermore, the molecules present in the early endosomes can either be reprocessed back to the plasma membrane of the endosomes or there can be incorporated into ILVs [18, 20]. These ILV transform the endosomes into multivesicular bodies (MVBs).

The generation of exosomes compels sorting of cellular components to the endosomal membrane and transport into early ILVs [19]. This is termed cargo sorting, where molecules are sorted into the ILVs, mediated by endosomal sorting complexes required for transport ESCRT-dependent and ESCRT-independent mechanisms [21, 22].

The ESCRT-dependent mechanism consists of 4 complexes and associated proteins. The ESCRT-0 complex which identifies the ubiquitylated proteins on the cytosolic side of the MVB membrane, segregates the proteins into micro domains, and then recruits the ESCRT-I complex, which in turn recruits ESCRT–II subunits. ESCRT-I and -II induce bud formation of the freshly generating ILVs within MVBs. The cytosolic RNAs and proteins have direct access into the interior of the forming vesicles at this stage, meanwhile, the ESCRT-II complex now recruits ESCRT-III subunits inside the neck of the freshly generated ILVs, hence resulting in their cleavage into free vesicles. Therefore, the main role of ESCRT-III is to drive vesicle cleavage. Finally, the free ubiquitin molecules together with the ESCRT subunits are then released into the cytosol for recycling of the ESCRT machinery [19, 23].

Certain proteins are sorted into ILVs in the absence of ESCRTs machinery, indicating the existence of alternative mechanisms of exosome biogenesis. Hence, cargo sorting can also occur through raft-based microdomains rich in sphingolipids. Sphingomyelinases can then act on the sphingolipids to release ceramide, which triggers ILV bud formation [19, 22].

The MVBs then follow either the secretory or degradative pathway. In the secretory pathway, the MVBs fuse with the plasma membrane, which results in the release of their internal vesicles called exosomes, into the extracellular space and the incorporation of the peripheral MVB membrane into the plasma membrane, meanwhile in the degradative pathway, the MVB fuse with lysosomes, in which the ILVs are degraded [24, 25].

The secretion of exosomes from MVBs is controlled by several Rab guanosine triphosphatase (GTPase) proteins, as these MVBs can either be degraded by lysosomes or fuse with the plasma membrane to be excreted [26, 27]. The Rab proteins involved in the movement of MVBs in the direction of the plasma membrane for exocytosis of exosomes are Rab11, Rab35, and Rab27 [26]. Nevertheless, the exact mechanism that directs MVBs towards the lysosomes instead of the plasma membrane for fusion remains indefinable [28], even though some studies have suggested the possible simultaneous presence of different MVBs subgroups in cells for which some are fated for degradation while others are fated for exocytosis [29]. Also, the regulation mechanisms responsible for exosome secretion are yet to be completely elusive. But some studies have demonstrated that the actin cytoskeletal regulatory protein cortactin plays an important role in regulating exosome secretion [30]. Exosomes are released into the extracellular space by different cell types and are extensively present in different bodily fluids/secretions, such as urine, blood [31], and sweat [32]. Their composition is based on the cell type from which they originate [16], and they are composed mainly of proteins, lipids, metabolites, mRNA, mitochondrial DNA, microRNA (miRNA) [33], and many other noncoding RNAs [34]. Most of this cargo is involved in the biogenesis and transportation ability of the exosomes [35]. Exosomes are dissimilar in their sizes and cargo, even when they originate from the same cell, but some partially common cargoes are present among exosomes even of different origins [36].

### 2.1. Physiological Roles of Exosomes

The function of exosomes is widely dependent on the cell or tissue of origin, as well as the availability of unique and specific proteins [37]. Their primary role is to enhance intercellular communication [38]. Exosomes have been extensively implicated in immune responses [23], angiogenesis [39], coagulation, cutaneous wound healing [40], transneuronal transport [41], tissue homeostasis, and morphogen transporters in the creation of polarity during development and differentiation [22, 23, 42]. The ability of exosomes to serve as intercellular communicators is a result of their cell-specific cargo of proteins, lipids, and nucleic acids. Studies have shown that exosomes released into the extracellular environment may influence the behavioral or phenotypic characteristics of both adjacent and/or distant cells away from their target cells [24]. Perhaps, they can also mediate the horizontal genetic material transfer via the surface adhesion molecules interactions [43]. Nevertheless, the cells which secrete exosomes can also reuptake their own secreted exosomes.

### 2.2. Pathological Roles of Exosomes

#### 2.2.1. Infectious Diseases

Exosomes can be hijacked by several infectious pathogens for survival and dissemination within the host. For instance, exosomes derived from virus-infected cells can deliver various pathogenic factors such as viral proteins and fragments of viral genome to other neighboring and distant cells. This exosome-facilitated delivery of pathogenic factors has been proven to modulate the immune responses to certain viral infections as well as modification of recipient cell response [44]. It has been shown that the Epstein-Barr virus (EBV) can suppress the expression of EBV-target genes in uninfected cells by transferring viral miRNAs to subcellular sites of gene repression in recipient cells through exosomes [45]. Other studies have demonstrated the role of exosomes in promoting HIV-1 infection via the transfer of co-receptors CCR5 and CXCR4 to recipient cells [12, 46]. As earlier mentioned, these nanovesicles can express several macromolecules including surface proteins that can facilitate the uptake of viral nucleic acids. In a recent study, it was revealed that exosomes significantly expressed the intracellular membrane protein, Reticulon 3 (RTN3), which modulated their uptake of replication-competent hepatitis C virus RNA and some viral nonstructural proteins [47]. This highlights the ability of exosomes to successfully incorporate infectious viral nucleic acids, serve as reservoirs for persistent infection or favor viral pathogenesis.

#### 2.2.2. Tumor Pathogenesis

Exosomes alter the content and behavior of the recipient cell through the transfer of bioactive molecules to recipient cells close to or distant from the original cells [14].

During tumor invasion, tumor-derived exosomes serve as an important messenger between the cancer and the microenvironment by facilitating cancer cell metastasis through the transfer of tumor suppressor miRNAs; this miRNA enters the recipient cells and target mRNA sequence, thereby inhibiting the expression of certain genes [48].

Reports have shown that exosomes containing miR-122 from cancer cells could suppress glucose uptake in nontumor cells in the premetastatic niche. This suggests the role of exosomes in programming cellular metabolism in the premetastatic niche, thereby facilitating disease progression accordingly [49]. Exosomes have also been shown to contribute to the survival and persistence of nutrient-deprived cancer cells by providing amino acids to them through a mechanism similar to that of micropinocytosis [50]. Owing to the ability of exosomes to transfer micro or mRNA and proteins to recipient neighboring tumor cells, they can also travel to distant sites and promote a protumor milieu to harbor metastatic niches [51].

### 2.3. Immunobiological Characteristics of Exosomes

The inherent property of exosomes in intercellular communication makes them an excellent shuttle for the transportation of several biochemical molecules capable of modulating a specific

response in its recipient cell. Moreover, the effects on the recipient cell can occur either by signaling through a receptor-ligand interaction or fusion with the plasma membrane to deliver vesicular contents into the cytosol of the recipient cell [52–54]. The cargo contents of the exosomes and its effect on the recipient cell are mediated by the microenvironment and the biological state of the parent cell. However, the differences in the stimuli for exosome biogenesis during different physiological and pathological states are not fully elucidated [55]. As indicated earlier, exosomes can express membrane adhesion molecules which are similar to that of their parent cell, thereby granting them access to their recipient cells. For example: the CD11c which is a specific marker of DCs can be expressed on DC-derived exosomes, MHC class I and II molecules, CD1, ICAM-1, and costimulatory molecules such as CD40 and CD86/CD80 [54]. The presence of MHC and costimulatory molecules on exosomes highlights the possibility of their involvement in antigen presentation and activation of immune cells to execute their effector functions. Like the classical professional antigen presenting cells (APCs), the process of antigen presentation by exosomes can either be direct or occur as a cross presentation. In direct presentation, the MHC–peptide complex on the exosomes is directly engaged by antigen-specific T cells, inducing T cells activation. In cross-presentation, APCs acquire exosome loaded antigens and further process them for T cell presentation and activation. All these immunological processes and features exhibited by exosomes explain the basis of protective or therapeutic benefits displayed by these nanovesicular bodies.

Exosomes can exert either a pro or anti-inflammatory reaction in the recipient cell depending on the signal derived from the parent cell. Very little is known about these dual effects of exosomes and so far, the results can be very different depending on the signal derived from the parent cell.

It has been reported that neutrophils can produce exosomes with immunoregulatory phenotypes, which decrease the release of proinflammatory cytokines and favor the release of TGF-*β*1 in LPS-activated DCs [56]. Results from other studies have shown that mycobacterial-infected murine macrophages were found to release infectious exosomes, which influenced DC migration. This led to an overall cross-talk between the innate and adaptive arms of the immune system. Thereby triggering the activation and proliferation of CD4+ and CD8+ T cells, including the activation of uninfected macrophages to produce cytokines and chemokines, which enhanced the transmigration of TCR-*β* + T cells [56]. Similarly, exosomes derived from professional antigen presenting cells such as DCs were shown to express both class I and II MHC molecules, certain adhesion, and costimulatory molecules that triggered the direct activation of CD4+ and CD8+ cells [57]. This suggests that even in the absence of activation signals from antigen presenting cells, a strong T cell response can still be generated through direct interaction with such DC-derived exosomes. Therefore, the ability of DC-derived exosomes to stimulate a strong T cell immune response depends on the presence of costimulatory molecules found on the surfaces of both the exosomes and DCs themselves. In connection to this, it has been reported that exosomes derived from cells lacking costimulatory molecules were not able to generate a strong T cell activation in the absence of DCs [56, 58]. The versatility of exosome functions provides possible explanations as to why they could be applied in infectious disease or cancer therapeutics. In fact, their ability to retain essential molecules from their parent cells also highlights their antitumor mediated activities. Studies have shown that exosomes from natural killer cells (NK cells) can retain cytolytic perforins and granzyme which have antitumoral activities [59].

Other studies have described the immunosuppressive potentials of some tumor derived exosomes

on NK cells. It has been demonstrated that tumor derived exosomes can suppress NK cells by regulating the expression of the NKG2D receptor [60]. These exosomes can promote regulatory T cells [61] expansion and favor the induction of T cells apoptosis through the activation of the Fas-Fas ligand pathway [62]. The mechanism through which Foxp3+ T regulatory (Treg) cells prevent inflammatory diseases is not fully understood. One of such mechanisms through which Treg cells use to suppress inflammation is through the use of Treg-derived exosomes. Treg-derived exosomes contained both premature and mature miRNAs. *In vitro* and *in vivo* transfer of miRNA-containing exosomes resulted in an increased suppression of Th1 cell proliferation and IFN-*γ* secretion [63]. Immature DCs were shown to secrete exosomes with similar anti-inflammatory properties as IL-10 treated DCs, which modulated the function of APCs and T cells *in vivo*, with subsequent reduction in inflammation [55].

Considering the immunobiological characteristics of exosomes and their role in shaping the immune response, it would be imperative to further harness these properties and foster their application in the field of oncology and infectious diseases.

### 2.4. Exosomes as Therapeutic Drug Delivery System

Over the years, liposomes and polymeric nanoparticles have been the preferred drug delivery systems. However, the ability of liposomes to evade the host immune system over a long circulating time and without toxicity remains to be explored. Polymeric nanoparticles on the other hand are more stable, but the biocompatibility and safety is still of major concern [64]. In recent years, the use of exosomes as potentially ideal drug delivery systems has gained momentum. This is due to the long circulating half-life, intrinsic ability to target tissues, biocompatibility, and little to no associated toxicity issues [65]. The exosome drug loading process can either be active or passive [66]. Active loading can be achieved by sonication, extrusion, electroporation, or drug conjugation techniques. This offers an enhanced loading efficiency and facilitates loading of larger molecules [67]. In passive loading, exosomes are incubated with the drug wherein the drug diffuses into the exosomes across a concentration gradient, or the drug is incubated with donor cells for uptake, which later secrete exosomes containing the drug [68]. Both approaches have been extensively explored in the context of infectious disease and cancer therapy. In this review, we described some studies involving the use of exosomes as drug delivery vehicles in infectious disease and cancer therapy.

### 2.5. The Role of Exosomes in Viral Pathogenesis and their Therapeutic Perspectives

As described earlier, exosomes have been shown to be key players in favoring viral persistence and dissemination. The ability of exosomes to execute their function of intercellular communication has made it possible for them to act as trojan-horses in viral dissemination [69]. Existing evidence has shown that exosome formation is similar to the sorting and budding of enveloped viruses. The majority of such viruses and other nonenveloped viruses [69] have been able to exploit/hijack these mechanisms [70] to package their viral components and secrete vesicles that are infectious and potentially less immunogenic, thereby establishing a persistent infection [69]. The variation in exosome content has also been shown to not only be influenced by its cell of origin but also disease conditions such as viral infections and cancer [70]. Some of the common exosome pathways hijacked by viruses include: ESCRT, Rab GTPases [70], and Tetraspanins apparatus [69]. Some viruses have been able to hijack the Rab GTPase pathway which plays a key role in directing the recycling of vesicles, maturation of early endosomes, formation of endolysosomes, and release of intraluminal vesicles (exosomes). Several negative strand RNA viruses have been able to hijack the short processing vesicular recycling mechanism (Rab 11) in favor of their infectious cycle [70]. This is correlated with the significant decrease in the virus infectious cycle seen when Rab 11 was depleted [70].

Another mechanism through which these viruses use exosomes in their favor includes packaging of their genetic material and proteins into multivesicular bodies. Considering that these ILV are formed by an invagination in the late endosome which takes in some of its cytosolic content into these vesicles. It is therefore possible that viruses shed their genetic content into the cytosol of the cells so as to be taken up by the ILV. Once this is established, it is possible that these viruses disrupt the different Rab GTPase to ensure their content is excreted out. For example, SeV, RSV, and IAV progeny RNA (in the form of viral ribonucleoproteins (vRNPs)) attach to Rab11 vesicles, facing the cytoplasm, to aid their transit to the plasma membrane's apical side [70]. HIV also uses the ESCRT pathway to facilitate budding [71].

The ability of certain viruses to hijack the different stages of exosome formation favors their pathogenesis and immune evasion. It has been shown that cellular uptake of exosomes packaged with viral proteins such as the HIV Nef protein can increase the susceptibility of nascent cells to HIV infection. Perhaps, HIV can package both its Nef protein and coreceptors like CCR5 and CXCR4 in exosomes and favor the infection of non-HIV targeted cells [72]. In addition, exosomes from HIV infected cells were able to transmit the transactivation response element (TAR) RNA, which places itself at the 5′ tail of HIV transcript copies and interacts with the Tat protein to produce viral RNAs. The TAR-RNA molecule generates miRNAs which suppress Bcl-2 interacting protein, ultimately promoting resistance to apoptosis and production of the virus [70]. Other studies have shown that exosomes derived from hepatitis B virus (HBV)-infected hepatocytes transport miR-21, miR-29a, and other miRNAs with immunoregulatory functions to THP-1 macrophages, which results in a downregulation of IL-12p35 mRNA expression in turn leading to a constrained host innate immune response [73].

The current evidence suggesting that viruses can hijack exosome sorting, loading, and excretion to foster their dissemination and create a persistent infection in their host raises a horde of questions on the true effectiveness of the current therapeutic measures against viral infections. Albeit this, there is also a window for improvement in the therapeutic measures especially in the treatment of viruses capable of establishing persistent infections such as HIV and Hep B and C.

It is evident that several viruses can hijack the intracellular machinery of exosomes to establish persistence in both target/nontarget cells. Therefore, a critical understanding of the various mechanisms involved in the uptake of viral proteins/nucleic acids by exosomes, and how these viral proteins regulate the intracellular machinery of recipient cells would be very imperative in the development of effective suppressive antitherapies against these viruses and also provide more insights on how viruses establish persistence in both target/nontarget cells.

### 2.6. Exosomes as Targets for Antiviral Therapy

The ability of exosomes to deliver antigens to target cells has made it possible for the development of virus-free vaccine. Perhaps, their anti-inflammatory, proangiogenic, and immunomodulatory characteristics have enabled scientists to further explore them for the development of effective immunomodulatory therapies. This could confer protection and prevent the exaggeration and increased inflammation seen in most virally infected patients especially in SARS-COV-2. EXOFlo, a bone marrow-derived exosomal-based product, was recently investigated for the treatment of severe COVID-19 in a nonrandomized open-label cohort study [74]. The ExoFlo was administered to twenty-four patients, and no side effects were observed after 72 hours. It was shown that this treatment strategy significantly improved on the patients' disease severity by restoring oxygen storage capacity, reducing cytokine storm, and boosting the immune response [74]. However, this is the first study of its sort to look into the therapeutic potential of MSC-derived exosomes for COVID-19.

Generally, exosomes derived from infected cells can deliver viral content to surrounding cells but can also induce antiviral immune responses [75]. Anticoli et al. identified a mutant of the Nef protein (Nef^mut^), a protein associated with raft micro domain at the cell membrane of CD4^+^ cells in HIV-1 virus [76]. This mutant was found to lack several anticellular properties typically displayed by the wild type Nef. They were able to engineer cells to release exosomes consisting of the Nef^mut^ protein; the engineered exosomes were taken up by professional APCs which presented the exosomes associated antigen. This led to the activation of specific cytotoxic lymphocyte immune response. They also demonstrated that the intramuscular inoculation of a DNA vector expressing HPV-E7 fused at the C-terminus of an exosome-anchoring protein led to the activation of antigen specific immunity which was powerful enough to counteract the growth of syngeneic tumor. They further tested the engineered exosomes incorporated with other viral antigens, such as those for HIV, Ebola, influenza, hepatitis B, hepatitis C, and West Nile viruses, and all the DNA vectors expressing viral antigens fused with Nef^mut^ elicited a CD8^+^ T cell specific immune response when injected in mice [76].

Exosomes provide a significant prospect and a novel therapeutic area for the delivery of different synthetic and biological molecules in disease therapy. The use of exosomes could provide the basis of antiviral therapy including the reduction of exaggerated inflammatory responses seen in most viral infections. Therefore, there is a need for further research to better understand and harness the therapeutic potentials of exosomes in antiviral therapy.

### 2.7. Exosomes as Targets for Anticancer Therapy

The biostability and abundance of exosomes has made it possible for them to be widely used in cancer immunotherapy and chemotherapy. Its use in Immunotherapy is widely achieved through the use of exosomes derived from pulsed DCs [77]. The purpose of cancer immunotherapy is to promote the antitumoral activity of cytotoxic T-lymphocytes (CTLs), assist in the initiation of tumor-specific CTLs in lymphoid organs, and establish effective and long-lasting anticancer immunity [78].

DC-derived exosomes have been used widely for immunotherapy against cancer where peptide-pulsed DCs can present antigens to T cells to induce their immune response. *In vivo* studies with mice models have shown that DC-derived exosomes contain MHC-peptide complexes and costimulatory molecules on their membrane, which enable them to prolong antigen presentation and boost immunization compared to antigen-presenting DCs. A study conducted by Lancaster and Febbraio showed that exosomes isolated from two MHC type-distinct mouse cell lines expressing tumor antigen human mucin 1 (hMUC1), induced an effective immune response, and suppressed hMUC1-expressing tumor cell growth in mice [79]. A similar outcome was achieved by Mahaweni et al., [80] who revealed that exosomes obtained from malignant mesothelioma cells were found to contain tumor antigens and that readministration of these exosomes could improve the overall survival of mesothelioma-bearing mice by activating antitumor immune responses [77]. Reports have also shown that in tumor-bearing mice vaccinated with Tumor Associated Exosomes-loaded DC, the TAEs were effectively ingested by DCs and subsequently upregulated the expression of CD11c, MHC II, and IL-12. This emphasizes the role of TAEs in stimulating a strong and specific immune response through DCs against its corresponding tumor, thereby reducing the side effects to cancer therapy [78]. Other studies have demonstrated the adjuvanticity of some tumor-derived exosome molecules such as the HSPs. It has been revealed that exosomes expressing the HSP-70 can activate an array of immune cells *in vivo* [81]. The adjuvant activity of these exosome-expressed molecules has been postulated to be the reason behind the very strong immune response observed after a single intraperitoneal injection of tumor peptide-loaded DC-derived exosomes in murine models, which led to a delay in tumor growth or to a complete tumor rejection [57]. The immunogenic potentials of HSP-70 have been further explored in several studies. Perhaps, it has been shown that recombinant human HSP-70 is capable of stimulating the production of IL-1*β*, TNF-*α*, IL-6, and IL-12 and can also promote the upregulation of costimulatory (CD86) and MHC II molecules in monocytes and dendritic cells [82, 83]. It has been hypothesized that the extracellular HSPs, released either as a result of cellular necrosis or via a stress-induced by the exocytotic pathway, may act as a potent danger signal to the immune system [84, 85]. Also, HSP70 renders tumor cells more sensitive to natural killer cell-mediated cytolytic attack [86]. There have also been great advancements with the use of exosomes as a delivery system for anticancer drugs to improve on the drug therapeutic effect and reduce side effects. Researchers have been able to engineer donor cells to obtain modified exosomes with particular receptors for better cell recognition since exosomes can recognize specific cell types through their surface receptors. For example, exosomes with Tspan8 can preferentially bind to CD11b and CD54-positive cells which possess a preferred method to synthetic nanoparticles which has low specificity delivery system because of their limited number of selective molecules that can be used for cell targeting [77]. It has been demonstrated that dendritic cells (DCs) engineered to express the *α*v integrin-specific iRGD peptide and Lamp2b fusion protein were able to secrete exosomes with the iRDG peptide on their surface. These engineered exosomes significantly increased drug delivery efficiency and antitumor effect on *α*v integrin-positive breast cancer cells in a mouse model [77].

Several studies have shown that anticancer drugs contained in exosomes have improved pharmacokinetic and pharmacodynamic properties, which boost their anticancer activity compared to when they are delivered as free drug molecules *in vivo*. [87]. In one study, mesenchymal cells derived from exosomes were loaded with paclitaxel (PTX), and its anticancer effects were evaluated against breast cancer. The results demonstrated that the engineered exosomes (PTX-NK-Exos) at the same dose of PTX dose had a higher inhibition rate on human breast cancer MCF-7 cells compared with free PTX. Interestingly, this phenomenon has been explained by some studies, and it has been found that the lipid bilayer of exosome can directly fuse to the cell membrane of recipient cells, thereby enhancing cellular internalization of PTX, leading to the release of the drug directly into the cytosol, hence improving the therapeutic effect [88, 89].

The combination of exosomes with some anticancer drugs has been shown to produce a better apoptotic outcome with an overall decrease in tumor progression. For instance, exosomes derived from a melanoma cell line engineered to have a tetracycline-regulated Survivin-T34A (a mutant of apoptotic inhibition protein Survivin) were plated on the pancreatic adenocarcinoma (MIA PaCa-2) cell line. The results showed that MIA PaCa 2 cells treated in culture using Survivin-T34A exosomes (1,500 *μ*g/mL) yielded a marked increase in apoptosis (30.5%). Also, the combination of exosomes containing Survivin-T34A with 10 *μ*M Gemcitabine displayed a greater enhanced apoptosis (32%) compared to 10% induced apoptosis when Gemcitabine was used alone [90]. Other studies have shown that the anticancer drug doxorubicin, when loaded with breast cancer-derived exosomes (ExoDOX) demonstrated more effectiveness than free doxorubicin in the treatment of breast cancer and ovarian cancer mouse models. In addition, the shuttling of doxorubicin inside exosomes increases drug stability and accumulation in the tumor, which ultimately improves treatment success [91].

Furthermore, loading of therapeutic nucleic acids such as small interfering (si) RNA into exosomes has also been shown to increase intracellular uptake by preventing nuclease induced degradation, thereby increasing the overall therapeutic effect compared with free RNA that is rapidly degraded by nucleases [87]. In one study, exosomes (iExosomes) derived from normal fibroblast-like mesenchymal cells were engineered to carry siRNA against oncogenic KRAS. iExosomes target oncogenic KRAS with greater efficiency. Interestingly, iExosomes harbored CD47, a “do not eat me” signal that prevents their nonspecific uptake by the reticuloendothelial system and which enables them to enter cells via micropinocytosis (an endocytic pathway that is exacerbated following oncogenic KRAS activation in cancer cells). The iExosomes treatment suppressed tumor growth in multiple mice models of pancreatic cancer, and the effects of tumor suppression were sustained for over 10 days after initial iExosome treatment was suspended, thus significantly increasing overall survival of the mouse [92, 93]. It should be noted that the oncogenic KRAS mutation is the most common cause of pancreatic cancer. It causes the KRAS protein to become permanently activated, acting as a molecular switch to activate several intracellular signaling pathways and transcription factors that promote cell proliferation, migration, transformation, and survival [93].

Considering the improved prognostic outcome seen with the use of cell derived exosomes in cancer drug therapy/immunotherapy, advanced research aimed at improving on the application of this therapeutic strategy could pave a gateway for the development of effective anticancer therapies.

### 2.8. Examples of Clinical Trials with Exosomes as Therapeutic Carriers

Several clinical studies have been conducted to elucidate the therapeutic potential of exosomes in patients suffering from certain infectious diseases or tumors. Recent studies from a phase 2 clinical trial showed that mesenchymal stem cell (MSC)-derived exosomes were able to reduce the excessive inflammatory reactions seen in severe COVID-19 patients. There was no evidence of prespecified inhalation-associated and severe adverse events or dose-relevant toxicity [94] The Gustave Roussy and Curie institutes have developed a lung cancer immunotherapy involving metronomic cyclophosphamide (mCTX) followed by vaccinations with tumor antigen-loaded dendritic cell-derived exosomes (Dex). mCTX was shown to break tolerance by inhibiting Tregs and restoring effector T cells and NK cells functions, while Dex was able to activate the innate and adaptive arms of the immune system. Although the Phase I trials showed a good safety profile and feasibility of the Dex vaccines, there was no induction of T cells in patients (NCT01159288). A similar study (phase II clinical trial) was conducted by Besse et al. to evaluate the clinical benefit of IFN-*γ*-Dex loaded with MHC class I and class II-restricted cancer antigens from patients with non-small-cell lung cancer (NSCLC). The findings of this phase II trial showed that (i) IFN-*γ*-Dex is a very well tolerated immunotherapy, (ii) effective clinical-grade IFN-*γ*-Dex production is feasible, and (iii) IFN-*γ*-Dex boosts NKp30-dependent NK cell functions while having no detectable induction of antigen-specific T cell responses when used as maintenance immunotherapy in chemotherapy-stabilized/responding NSCLC patients [95].

Despite some successful outcomes seen in clinical trials with exosomes as therapeutic carriers, the problem of poor quality control and no gold standard method for large-scale exosome purification still remain a major issue. Furthermore, the purification procedure of exosomes might raise some challenges such as contamination during clinical-grade exosome isolation that can affect clinical trial integrity and results. As a result, a novel exosome purification technology for therapeutic use is required [96].

## 3. Conclusion and Perspectives

Exosomes play a key role in intercellular communication and are excellent shuttle for the transportation of several biochemical molecules capable of modulating a specific response in its recipient cell. These nanovesicles have been shown to play a pivotal role in cancer therapy by improving on the suppressive effects of loaded anticancer drugs. Despite their role in viral persistence and dissemination, they have been shown to provide a basis for antiviral therapy including the reduction of exaggerated inflammatory responses seen in most viral infections. Therefore, there is a need for further research to better understand and harness the therapeutic potentials of exosomes in antiviral therapy. Also, considering the improved prognostic outcome seen with the use of cell-derived exosomes in cancer drug therapy/immunotherapy, advanced research aimed at improving on the application of this therapeutic strategy could pave a gateway for the development of effective anticancer therapies.

## Data Availability

All data are included within the article.

## Abbreviations

ESCRT: Endosomal sorting complexes required for transport
GTPase: Guanosine triphosphatase
miRNA: microRNA
LPS: Lipopolysaccharide
TAR: Transactivation response element
hMUC1: Antigen human mucin 1
TAEs: Tumor associated exosome
PTX: Paclitaxel
PTX-NK-Exos: Paclitaxel natural killer cell-derived exosome complex
ExoDOX: Cancer-derived exosome loaded with doxorubicin
siRNA: Short interfering RNA
SARS-COV-2: Severe acute respiratory syndrome coronavirus 2.
